# Alkylation of rabbit muscle creatine kinase surface methionine residues inhibits enzyme activity in vitro

**DOI:** 10.1007/s00204-021-03137-6

**Published:** 2021-08-16

**Authors:** Dirk Steinritz, Robin Lüling, Markus Siegert, Harald Mückter, Tanja Popp, Peter Reinemer, Thomas Gudermann, Horst Thiermann, Harald John

**Affiliations:** 1 Bundeswehr Institute of Pharmacology and Toxicology, Neuherbergstraße 11, 80937 Munich, Germany; 2 Walther-Straub-Institute of Pharmacology and Toxicology, Ludwig-Maximilians-Universität Munich (LMU), Goethestraße 33, 80366 Munich, Germany; 3 Bundeswehr Institute of Radiobiology, Neuherbergstraße 11, 80937 Munich, Germany; 4 Present Address: Bundeswehr Medical Service Academy, Ingolstädter Straße 240, 80939 Munich, Germany; 5 Present Address: Proteros Biostructures GmbH, Bunsenstraße 7a, 82152 Planegg, Germany; 6 Present Address: AM1 Ventures GmbH, Fasanenstraße 27a, 81247 Munich, Germany

**Keywords:** Enzyme activity, Free cysteine residue, Mass spectrometry, Alkylating agents, Methionine, Sulfur mustard, Hydroxyethylthioethyl

## Abstract

Creatine kinase (CK) catalyzes the formation of phosphocreatine from adenosine triphosphate (ATP) and creatine. The highly reactive free cysteine residue in the active site of the enzyme (Cys^283^) is considered essential for the enzymatic activity. In previous studies we demonstrated that Cys^283^ is targeted by the alkylating chemical warfare agent sulfur mustard (SM) yielding a thioether with a hydroxyethylthioethyl (HETE)-moiety. In the present study, the effect of SM on rabbit muscle CK (rmCK) activity was investigated with special focus on the alkylation of Cys^283^ and of reactive methionine (Met) residues. For investigation of SM-alkylated amino acids in rmCK, micro liquid chromatography-electrospray ionization high-resolution tandem-mass spectrometry measurements were performed using the Orbitrap technology. The treatment of rmCK with SM resulted in a decrease of enzyme activity. However, this decrease did only weakly correlate to the modification of Cys^283^ but was conclusive for the formation of Met^70^-HETE and Met^179^-HETE. In contrast, the activity of mutants of rmCK produced by side-directed mutagenesis that contained substitutions of the respective Met residues (Met^70^Ala, Met^179^Leu, and Met^70^Ala/Met^179^Leu) was highly resistant against SM. Our results point to a critical role of the surface exposed Met^70^ and Met^179^ residues for CK activity.

**Supplementary Information:**

The online version contains supplementary material available at 10.1007/s00204-021-03137-6.

## Introduction

Creatine kinase (CK, EC 2.7.3.2) belongs to an evolutionarily conserved group of enzymes. It catalyzes the reversible, magnesia-catalyzed (Mg) reaction between creatine (Cr) and adenosine triphosphate (ATP) forming phosphocreatine (PCr) and adenosine diphosphate (ADP) according to the reaction MgATP + Cr ⇆ MgADP + PCr (Schlattner et al. 2016), thus being a key player in maintaining cellular energy homeostasis. Four major CK isozymes, two cytosolic and two mitochondrial, which form dimers and octamers, respectively, have been described (McLeish and Kenyon 2005). The cytosolic subunits can be either *B* type (brain) or *M* type (muscle) resulting in either CK-MM, CK-BB or CK-MB dimers (Bais and Edwards 1982; Wallimann et al. 2011). The individual CK isoenzymes are encoded by four independent genes (Qin et al. 1998; McLeish and Kenyon 2005) with an overall conservation of the amino acid sequence between 78% to more than 99% and a highly preserved active site (Mühlebach et al. 1994; Qin et al. 1998; McLeish and Kenyon 2005). The first CK crystal structure was solved in 1996 (Fritz-Wolf et al. 1996). Since then, additional high-resolution crystal structures of different CK subtypes from various species were reported that allowed the assignment of enzyme function to certain amino acid motifs (Rao et al. 1998; Eder et al. 1999, 2000a; Bong et al. 2008). Several amino acid residues, including cysteine (Cys) (Reddy and Watts 1978; Furter et al. 1993; Reddy et al. 2000; Wang et al. 2006), arginine (Wood et al. 1998), histidine (Forstner et al. 1997) as well as tryptophan and aspartic acid (Gross et al. 1994; Cantwell et al. 2001) are considered to be important for substrate binding and conversion (Bickerstaff and Price 1978; Eder et al. 2000b). In particular, the highly reactive Cys^283^ residue (amino acid numbering does include the N-terminal methionine (Met) residue) in the active site of the enzyme is discussed to play a pivotal role in this context (Maggio and Kenyon 1977; Bickerstaff and Price 1978; Furter et al. 1993; Reddy et al. 2000; Wang et al. 2006) and is subject of current research.

It was shown that alkylation of CK with iodoacetamide (IAA) or iodomethane, which both target free Cys residues, reduced the enzyme activity (Atherton et al. 1970; Reddy and Watts 1978). Treatment of CK with the alkylating chemical warfare agent sulfur mustard (SM) resulted in the formation of the specific hydroxyethylthioethyl-(HETE)-moiety at Cys^283^ (Lüling et al. 2021; Steinritz et al. 2021) but its effect on enzyme activity has not been addressed so far. This was investigated in the present study with particular focus on Cys^283^ but also on reactive Met residues, that in principle might also be essential for the activity of certain enzymes (Rogers et al. 1976) and were shown to be a potential target of alkylation by SM (Siegert et al. 2019).

## Materials and methods

### Chemicals

SM and its eight-fold deuterated analog d8-SM (purity of SM and d8-SM > 99%, assessed in-house by nuclear magnetic resonance, NMR) were made available by the German ministry of Defense. Rabbit muscle CK (rmCK), IAA, ethanol (EtOH), 4-(2-hydroxyethyl)-piperazine-1-ethanesulfonic acid (HEPES), NaOH, 25% (w/v) polyethyleneglycol 4000, ammonium acetate, dithiothreitol (DTT), glycerol, sodium citrate, dimethylformamide, ethylenediaminetetraacetic acid (EDTA), ethyleneglycoltetraacetic acid (EGTA), Na_2_HPO_4_, NaH_2_PO_4_, protease-inhibitor mix, nuclease mix, and Tween20 were obtained from Sigma-Aldrich (Steinheim, Germany). HCl and NaCl were from Carl Roth (Karlsruhe, Germany) and phosphate-buffered saline (PBS) was from Life Technologies (Gibco, Karlsruhe, Germany). CK activity assay kit was purchased from Abcam (Cambridge, UK). Proteinase K (ProtK), water (LC–MS grade), acetonitrile (ACN, LC–MS grade) and formic acid (FA, > 98%) were purchased from Merck (Darmstadt, Germany). NH_4_HCO_3_ (ultra-grade, 99.5%) was from Fluka (Buchs, Switzerland) and three-fold deuterated atropine (d3-atr) from CDN Isotopes (Pointe Claire, Quebec, Canada).

### CK activity assay

The effect of SM on rmCK enzyme activity was examined using a CK activity assay. RmCK was dissolved in PBS (pH 7.4, 10 mg/mL). This solution (49 µL) was mixed with 1 µL SM solution (200 mM in EtOH) in a 96-well plate with clear flat bottom (Greiner Bio-One, Frickenhausen, Germany) and incubated for 0, 5, 10, 15, 20, 25, and 30 min before starting the assay. After the respective incubation periods, 50 µL of the incubation solution was mixed immediately with 34 µL CK assay buffer, 2 µL CK enzyme mix, 2 µL CK developer, 2 µL ATP solution and 10 µL CK substrate solution (all included in the assay). The optical density (OD) at 450 nm was measured every minute for a total of 60 min at 25 °C using the TECAN infinite M200 PRO photometer (TECAN, Crailsheim, Germany). To characterize the effects on enzyme activity caused by the solvent or ${\mathrm{Cl}}^{-}$ as one hydrolysis product of SM, or ${\mathrm{H}}^{+}$, that is released during the alkylation reaction, rmCK was incubated with 1 µL of either EtOH or 8 mM NaCl or 0.2 mM HCl and measurements were performed as described above. The NaCl concentration of 8 mM mimics the theoretical maximum concentration of ${\mathrm{Cl}}^{-}$ resulting from complete hydrolysis of 4 mM SM.

Enzyme activities of mutant rmCK variants were analyzed after 15 min of incubation with different concentrations of SM (final concentrations: 0.04, 0.4, 1, 4, 10, 20, or 40 mM SM) using the same procedure.

### µLC-ESI MS/HR MS analysis

#### Sample preparation

For investigation of SM-alkylated amino acids in rmCK, micro liquid chromatography-electrospray ionization high-resolution tandem-mass spectrometry (µLC-ESI MS/HR MS) measurements were performed using the Orbitrap technology. For this purpose, 198 µL of rmCK solution (10 mg/mL in PBS) was mixed with 2 µL solution of either ethanolic SM or d8-SM solution (final concentrations: 0.04, 0.4, 1, 4, 10, 20, and 40 mM, each) at room temperature (RT) for 15 min in an ultrafiltration (UF)-device (molecular weight cut-off, MWCO, 10 kDa, Amicon, Merck-Millipore, Darmstadt, Germany). After washing with 300 µL water and UF (15 min, 25 °C, 9,770 RCF), samples were diluted with PBS to a rmCK concentration of 2 mg/mL. Subsequently, 200 µL of each sample was incubated with 2 µL DTT solution (10 mg/mL in water) for 1 h at RT following incubation with 2 µL IAA solution (10 mg/mL in water) for 45 min at RT. Next, samples were subjected to UF (15 min, 25 °C, 9,770 RCF) and the retentate was diluted with PBS to a final rmCK concentration of 2 mg/mL. For proteolysis, 100 µL of each sample was incubated with 100 µL ProtK solution (15 mg/mL in 50 mM NH_4_HCO_3_) and 300 µL 50 mM NH_4_HCO_3_ buffer in an UF-device at 50 °C for 2 h. Filtrates obtained from subsequent UF were diluted 1:3 with d3-atr solution (3 ng/mL in 0.5% v/v FA) prior to µLC-ESI MS/HR MS.

#### Chromatographic separation

For chromatographic separation of 20 µL sample volume, a MicroPro pump (Eldex Laboratories, Napa, CA, USA) was used in combination with an INTEGRITY autosampler (sample tray kept at 15 °C) equipped with a 20 µL sample loop and a MISTRAL column oven (both Spark Holland, Emmen, The Netherlands). The chromatographic system was controlled by the Eldex MicroPro 1.0.54 control software (Eldex Laboratories). Samples were separated on an ACQUITY UPLC HSS T3 column (C18, 50 × 1.0 mm I.D., 1.8 µm, 100 Å, Waters, Eschborn, Germany) protected by a security guard ultra-cartridge (C18-peptide, Phenomenex, Aschaffenburg, Germany) with two gradients of solvent A (0.05% v/v FA) and solvent B (ACN/H_2_O 80:20 v/v, 0.05% v/v FA): µLC-gradient 1: *t* [min]/B [%]: 0/0; 3/0; 35/40; 35.5/95; 39.5/95; 40/0 including an initial equilibration period of 5 min and µLC-gradient 2: *t* [min]/B [%]: 0/0; 12/35; 12.5/95; 14.5/95; 15/0 including an initial equilibration period of 5 min.

#### MS/HR MS analysis

For identification and relative quantification of alkylated peptides originated from SM- and d8-SM-treated (40 mM, each) rmCK after cleavage with ProtK, a Q Exactive Plus (ThermoFisher Scientific, Waltham, USA) Orbitrap mass spectrometer was used. It was coupled online to the µLC system via the HESI-II probe. All MS experiments were performed in the positive mode using the following settings: sheath gas flow 23 arbitrary units (a.u.), auxillary (aux) gas flow 8 a.u., sweep gas flow 1 a.u., spray voltage 3.5 kV, capillary temperature 250 °C, S-lens RF level 50 a.u. and aux gas heater temperature 125 °C. The Orbitrap instrument was calibrated daily using the Pierce™ LTQ ESI positive ion calibration solution (ThermoFisher Scientific) according to the manufacturer’s protocol.

For identification of rmCK-derived alkylated peptides, data-dependent tandem-mass spectrometry (ddMS^2^) scans (top 10) in combination with µLC gradient 1 were used. A full-MS survey scan in the range of *m/z* 200–*m/z* 1000 was performed with a resolution of 70,000 full width at half maximum (FWHM), with an automatic gain control (AGC) target of 3 × 10^6^ charges and a maximum injection time (IT) of 200 ms. For highest mass accuracy, the ubiquitous softener bis(2-ethylhexyl)terephthalate (single protonated, *m/z* 391.28429) was used as a lock mass. Identified precursor masses, which met the ddMS^2^ settings (minimum AGC target: 1 × 10^3^ charges; intensity threshold: 1 × 10^4^ charges; charge exclusion: *z* ≤ 4; peptide match: preferred; exclude isotopes: on) were measured by MS^2^ scans (17,500 FWHM; AGC target: 1 × 10^5^ charges; maximum IT: 100 ms; loop count: 10; MSX count: 1; isolation window: ± 2 Th; fixed first mass: *m/z* 100; normalized collision energy (NCE): 25 V). In addition, peptides also showing precursor masses with the d8-mass shift (4 ppm tolerance interval) and similar retention times (*t*_R_, ± 0.5 min) were fixed and automatically assigned to SM-alkylated peptides derived from rmCK (2 ppm mass tolerance).

For relative quantification of alkylated peptides including ThrCys^283^(-HETE)ProSer (TC^283^(-HETE)PS), IleMet^70^(-HETE)ThrVal (IM^70^(-HETE)TV), LysSerMet^179^(-HETE)ThrGlu (KSM^179^(-HETE)TE) as well as of the internal standard d3-atr, parallel reaction monitoring (PRM) was performed in combination with µLC gradient 2. For all experiments, the resolution of the Orbitrap mass analyzer was set to 17,500 FWHM, the AGC target to 2 × 10^5^ charges, the maximum IT to 80 ms, and the isolation window for precursor masses to Δ*m/z* ± 2.0. Additional MS parameters depending on the analyte detected are summarized in Table 1.

**Table 1:** MS parameters used for the detection of rmCK-derived peptides adducted by SM

Analyte	Precursor ion species	Precursor ion [*m/z*]	Product ion [*m/z*]	CE [V]
TC^283^(-HETE)PS	[M + H]^+^	511.2	137.008	33
IM^70^(-HETE)TV	[M]^+^	567.3	105.037	25
KSM^179^(-HETE)TE	[M + H]^2+^	350.2	105.037	25
d3-atr	[M + H]^+^	293.2	127.131	42

*CE* collision energy, *d3-atr* triple deuterated atropine, *HETE* hydroxyethylthioethyl-moiety

#### Calculation of alkylation ratios

For calculation of the relative extent of alkylation by SM or d8-SM, the peak areas obtained from µLC-ESI MS/HR MS analysis of the corresponding non-alkylated peptides comprising IM^70^TV, KSM^179^TE and TC^283^PS were determined. Peak areas found after incubation with SM were related to those obtained without incubation to calculate the relative ratio of non-modified amino acids. By subtracting the latter values from 100%, the corresponding alkylation ratios were obtained.

### Site-directed mutagenesis

For functional analysis of the residues Met^70^ and Met^179^ in rmCK, two recombinant single mutants (Met^70^Ala and Met^179^Leu) and one recombinant double mutant (Met^70^Ala/Met^179^Leu) were produced (Proteros biostructures, Martinsried, Germany). Three plasmids inducing the desired mutations were synthesized by Geneart (Regensburg, Germany): (i) HIS6-TEV-rabbitCK(1–381)-Met179Leu, (ii) HIS6-TEV-rabbitCK(1–381)-Met70Ala, and (iii) HIS6-TEV-rabbitCK(1–381)-Met70Ala/Met179Leu. Plasmids were transformed in *E. coli* NiCo21(DE3) obtained from New England Biolabs (Ipswich, MA, USA) for rmCK protein expression.

#### Protein expression and purification

*E. coli* NiCo21(DE3), transformed with wildtype rmCK (wt-rmCK) and rmCK mutant expression plasmids as described above, were shaken in terrific broth (TB)-medium (in-house preparation) at 37 °C. Expression was induced by adding 1 mM isopropyl ß-D-1-thiogalactopyranoside (Sigma-Aldrich, Taufkirchen, Germany). Three hours after induction, bacterial cells were harvested by centrifugation. Wt-rmCK and mutant proteins (Met^70^Ala, Met^179^Leu, Met^70^Ala/Met^179^Leu) were purified from the soluble fraction by Ni–NTA (nickel-nitrilotriacetic acid) affinity chromatography (Sigma-Aldrich). The N-terminal His6-fusion tag was removed by cleavage with tobacco etch virus (TEV) protease (in-house preparation) and wt-rmCK and mutant proteins were subsequently further purified to apparent homogeneity using size exclusion chromatography. The different rmCK proteins were lyophilized for storage.

### Statistical analysis and graphical output

The statistics software *R* (R Core Team 2020) with the graphical user interface *RStudio* (version 1.1.383) (RStudio Team 2020) was used for statistical analysis and graphic presentation. The R package *drc* (Ritz et al. 2015) was used for non-linear curve fitting (four-parameter log-logistic function; LL.4). Group differences were calculated by using Student’s *t* test included in the *ggpubr* (Kassambara 2020) package. Graphical output was done by using *ggplot* from the *tidyverse* package (Wickham et al. 2019).

## Results and discussion

Although various CK isoforms and their transition states revealed some insights into the communication between the subunits, substrate binding and conversion (Rao et al. 1998; Eder et al. 1999, 2000a; Lahiri et al. 2002), the exact catalytic mechanism of CK is not completely understood (Ohren et al. 2007; Shen et al. 2001; Tisi et al. 2001). CK possesses one highly reactive sulfhydryl-group (free cysteine residue) per subunit. This group can be modified by a number of sulfhydryl-specific reagents (Kenyon and Reed 1983), with impact on enzyme activity (Mahowald 1965; Buechter et al. 1992). This residue has been identified as Cys^283^ in the primary structure of rmCK (Mahowald 1965) and is conserved in all known CK sequences of other species (Buechter et al. 1992). Hence, rmCK (UniProtKB P00563) was used in the present study instead of human muscle CK (hmCK, UniProtKB P00563) due to economic reasons. Alignment of rmCK and hmCK using the Clustal Omega program (Sievers et al. 2011) revealed an overall identity of the amino acid sequences of 96.6% with a complete matching of all Cys and Met positions. Cys^283^ was recently identified as a target of alkylating agents including SM (Lüling et al. 2021; Steinritz et al. 2021). As Cys^283^ in the active site of CK (Wang et al. 2006) is supposed to bind creatine by electrostatic interactions between the free thiol-moiety of its side chain and the guanidine-group of creatine (Bong et al. 2008), we assumed that a loss of CK activity is due to the alkylation of this residue after treatment with SM.

### CK activity after alkylation by SM

**Fig. 1:**
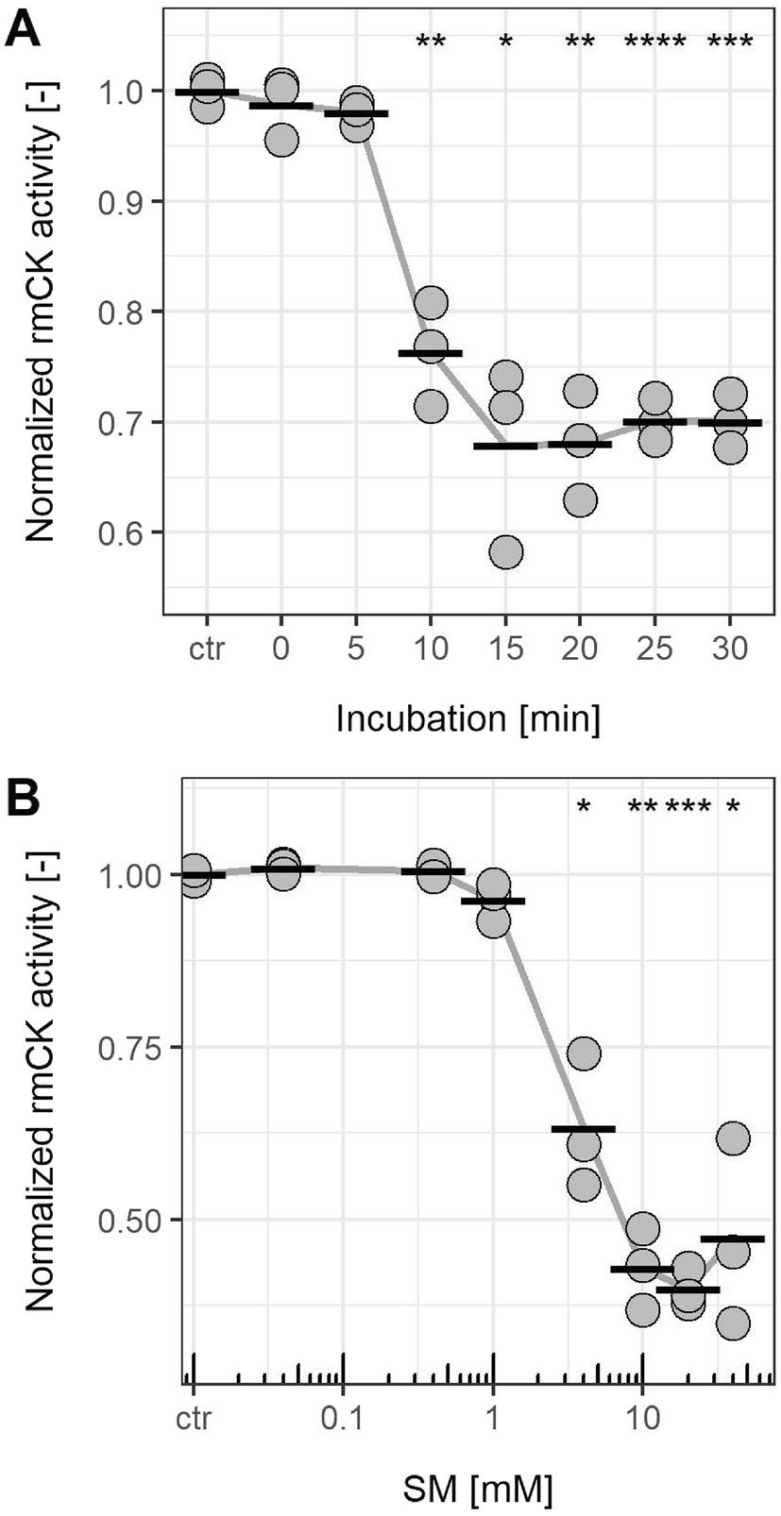
Creatine kinase activity after alkylation by SM. Enzyme activity of rabbit muscle creatine kinase (rmCK) was assayed using a colorimetric assay to monitor ATP conversion as area under the curve (AUC). Normalized activity was calculated from values of the respective AUC of non-treated rmCK control (ctr, activity 1.0). **A** RmCK was incubated with 4 mM SM for different times before starting the activity measurements. **B** Activity of rmCK after incubation with different SM concentrations for 15 min, each. AUC normalized to control levels is illustrated. Asterisks indicate significant differences (* < 0.05, ** < 0.01, *** < 0.001, **** < 0.0001) between the control group and time (**A**) or ctr and SM concentration (**B**). Data are derived from 3 independent experiments (*n* = 3)

Treatment of rmCK with 4 mM SM for at least 10 min revealed a significant decrease of the enzyme activity compared to the non-treated enzyme, reaching its minimum after 15 min (Fig. 1A). Therefore, a 15 min incubation period with SM was chosen for the subsequent dose–response experiments.

SM concentrations ≥ 4 mM significantly reduced the rmCK activity (Fig. 1B). In contrast, the solvent (2% v/v EtOH) or the SM reaction (${\mathrm{H}}^{+}$) and hydrolysis products (${\mathrm{Cl}}^{-}$) did not affect rmCK activity (Suppl. Fig. S1) thus confirming that alkylation caused the reduced enzyme activity. This is in accordance to previous studies reporting the loss or at least the decrease of CK activity after the treatment with alkylating IAA (Price 1979; Fossel and Hoefeler 1987).

### Identification of alkylated Cys^283^ after SM treatment of rmCK and correlation to enzyme activity

**Fig. 2:**
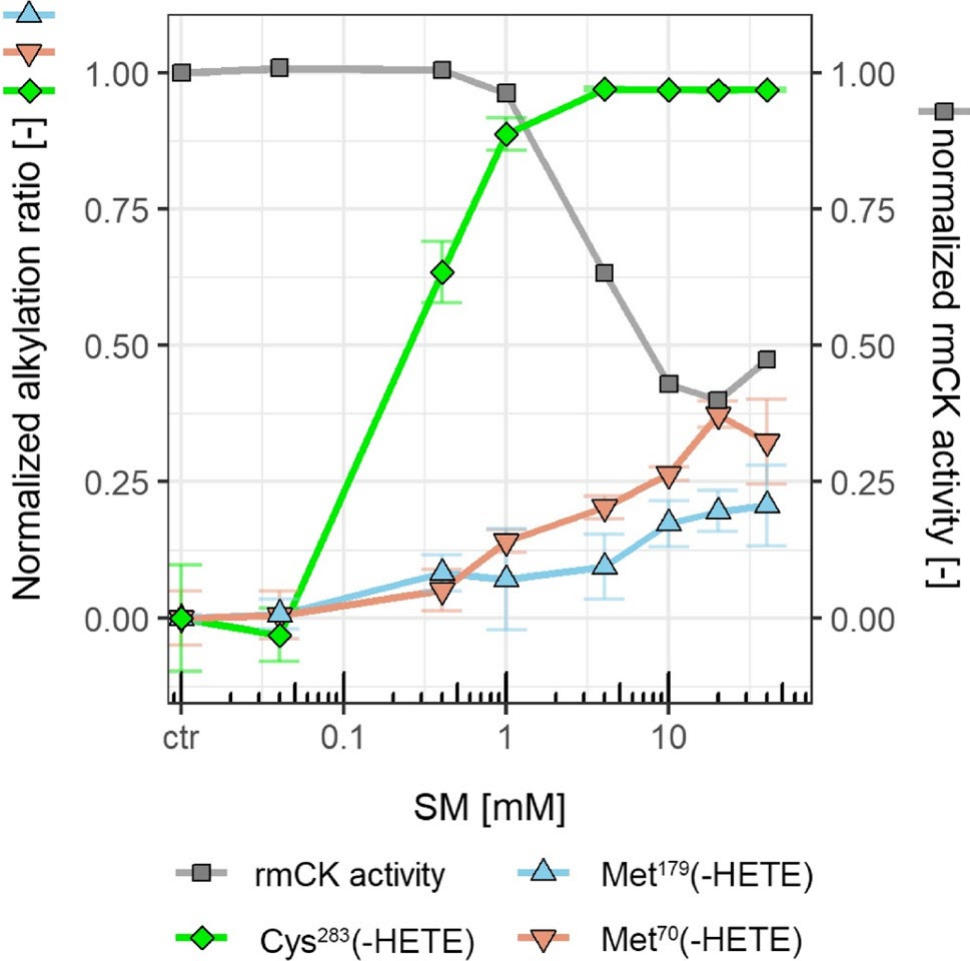
Correlation between alkylation ratios of Cys^283^, Met^70^, Met^179^ and rmCK activity. After 15 min treatment of wt-rmCK with different SM concentrations indicated, alkylation ratios (green line and diamonds for Cys^283^, red line & downwards triangles for Met^70^, and blue line and upright triangles for Met^179^) were calculated after µLC-ESI MS/HR MS analysis by normalizing the peak area of the respective precursor ions to control levels of non-treated rmCK. The rmCK activity is given as normalized AUC (squares with dashed gray line). Normalized alkylation ratios are given as green line and diamonds for Cys^283^(-HETE), red line and downward triangles for Met^70^(-HETE), and blue line and upward triangles for Met^179^(-HETE). The error bars display the standard deviation obtained from 3 independent experiments (*n* = 3) (color figure online)

We succeeded in the detection of diverse peptides comprising all cysteine residues (Cys^74^, Cys^146^, Cys^254^, and Cys^283^) of rmCK after proteolysis with ProtK by µLC-ESI MS/HR MS analysis (data not shown). Cys^283^ is located in the active site of CK and is susceptible by chemical modifications (Reddy et al. 2000). After treatment of rmCK with SM, alkylated Cys^283^ was detected present in the protonated tetrapeptide TC^283^(-HETE)PS ([M + H]^+^ *m/z* 511.2) which is characterized by its product ions at *m/z* 105.037 and *m/z* 137.009 obtained by MS/HR MS (Lüling et al. 2021; Steinritz et al. 2021). Alkylation of Cys^74^, Cys^146^, and Cys^254^ only occurred to a negligible extent (data not shown). After treatment with 1 mM SM, Cys^283^ was found to be alkylated to approx. 90% but rmCK activity was almost not affected (Fig. 2). Therefore, we concluded that alkylation of Cys^283^ by SM has no major impact on rmCK activity.

### Identification of alkylated Met^70^ and Met^179^ after SM treatment of rmCK and correlation to enzyme activity

RmCK contains 10 Met residues in total (Met^30^, Met^70^, Met^179^, Met^207^, Met^240^, Met^246^, Met^272^, Met^360^, Met^363^, and Met^376^). Following proteolysis of rmCK with Prot K, diverse peptides were identified by µLC-ESI MS/HR MS that contained these residues thus allowing monitoring of their chemical modification after SM treatment. The most prominent peptides proven to be alkylated at Met^70^ and Met^179^ are summarized in Table 1 with respect to their mass spectrometric detection.

Met residues in general represent important regulators of protein function and enzyme activity (Taylor et al. 2018; Aledo 2019; Lim et al. 2019; Valley et al. 2012). They interact with aromatic amino acid side chains (e.g. phenylalanine, tyrosine, tryptophan) thereby significantly increasing the stability of proteins (Valley et al. 2012). Met residues contain a single nucleophilic sulfur atom in the side chain that is accessible for covalent modifications. After alkylation, a Met sulfonium ion is formed containing a permanent positive charge at the sulfur atom (Kramer and Deming 2012) which may cause conformational changes in the protein tertiary structure or may even cause disturbance of the protein secondary structure (Kramer and Deming 2013). Recently, it was shown that Met residues may also be alkylated by SM as proven for Met^329^ in human serum albumin (Siegert et al. 2019).

The extent of SM-induced alkylation of Met^272^, Met^360^ and Met^376^ did not correlate with the impaired enzyme function (Suppl. Fig. S3). In contrast, the alkylation ratios of Met^70^ and Met^179^ increased in a concentration-dependent manner and the rmCK activity decreased reciprocally (Fig. 2). Considering the described three-dimensional structure of rmCK, these Met residues are exposed to the surface and thus allow a good accessibility by SM. In contrast, Met^272^, Met^360^ and Met^376^ are in positions of impaired accessibility which explain their low extent of alkylation. Therefore, our results suggest a causal relationship between Met^70^ and Met^179^ and enzyme activity. To prove this hypothesis, relevant mutants of rmCK were generated and their susceptibility towards SM was evaluated.

### Activity of rmCK mutants (Met^70^Ala, Met^179^Leu) after SM treatment

To elucidate the role of Met^70^ and Met^179^ with regard to enzyme activity in more detail, rmCK mutants (Met^70^Ala, Met^179^Leu and Met^70^Ala/Met^179^Leu) were generated by recombinant expression. Ala or Leu were chosen as substitute amino acids because they preserve the environmental characteristics in the protein structure as close as possible albeit they cannot be alkylated. As analyzed from the database-accessible structure, the environment of Met^70^ in wt-rmCK is mainly hydrophilic, while that of Met^179^ is mainly hydrophobic. Therefore, Ala was used for substitution of Met^70^ and Leu for Met^179^. Mass spectrometric analysis (ddMS^2^) of the three mutants and the wt-protein after proteolysis using trypsin demonstrated the integrity of the four proteins (Suppl. Fig. S2A).

**Fig. 3:**
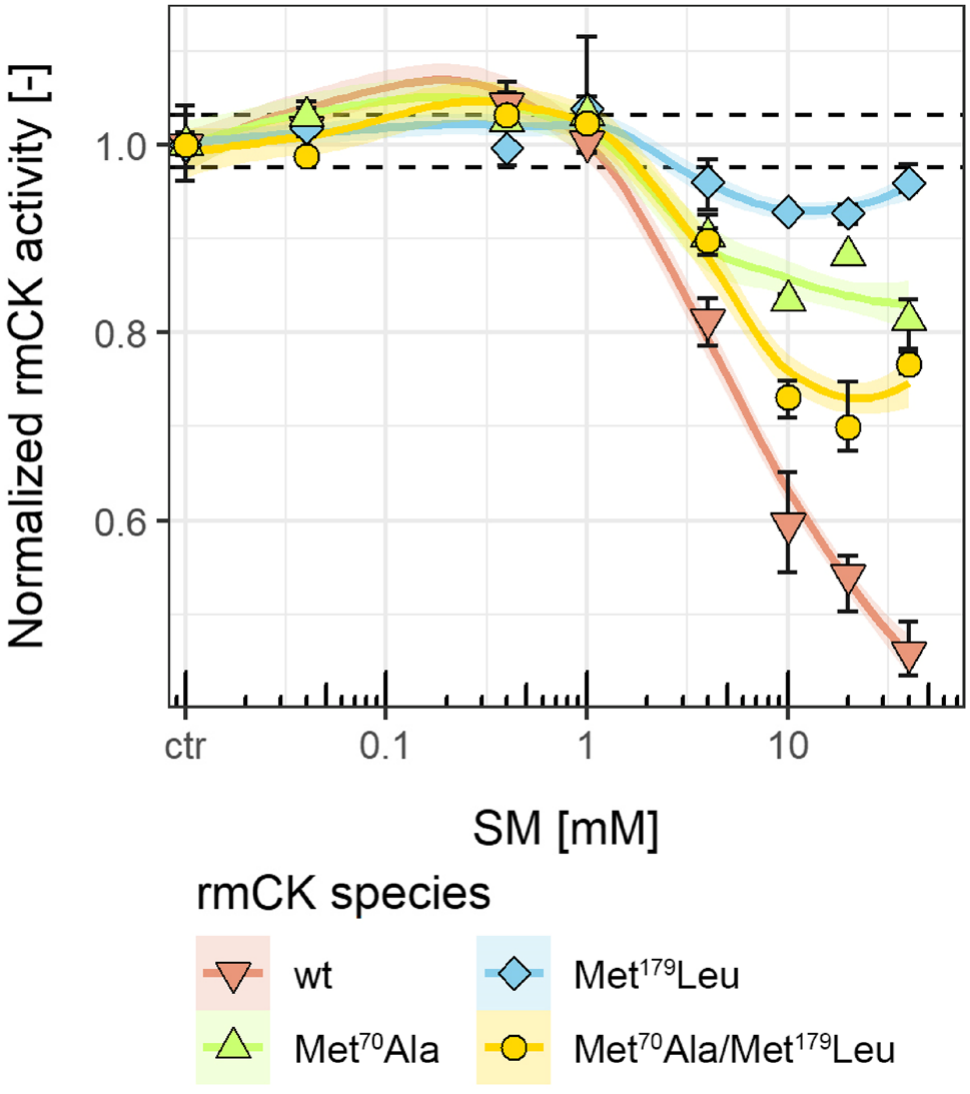
Activity of wt-rmCK and rmCK mutants after SM treatment. Wt-rmCK and rmCK mutants (Met^70^Ala, Met^179^Leu and Met^70^Ala/Met^179^Leu) were incubated for 15 min with different SM concentrations indicated. Activity of rmCK was colorimetrically (450 nm) assayed using a commercial test kit monitoring ATP conversion. Normalized rmCK activity is illustrated as area under the curve (AUC) calculated from 3 independent experiments (*n* = 3). The error bars display the standard deviation and ribbons represent the 95% confidence intervals of the respective curve fits. Red line and downward triangles represent wt-rmCK, green line and upward triangles represent Met^70^Ala, blue line and diamonds represent Met^179^Leu, and yellow line and circles represent Met^70^Ala/Met^179^Leu. Activity levels (mean ± SD) of non-treated wt-rmCK are indicated by dotted lines (color figure online)

All mutants exhibited a relative mean enzyme activity (*n* = 3) similar to that of wt-rmCK (wt-rmCK: 1.0 ± 0.015, Met^70^Ala: 0.916 ± 0.077, Met^179^Leu: 1.0 ± 0.011 and Met^70^Ala/Met^179^Leu: 0.964 ± 0.057) thus indicating that the related non-modified Met residues are not essential for the enzyme function. In contrast, after treatment of the enzymes with SM, the activity of the mutants was obviously less diminished than that of the wt-rmCK showing remaining activities after incubation with 40 mM SM of 0.46 ± 0.03 for wt-rmCK, 0.81 ± 0.03 for Met^70^Ala, 0.96 ± 0.02 for Met^179^Leu, and 0.77 ± 0.01 for Met^70^Ala/ Met^179^Leu (Fig. 3). Especially, the activity of the Met^179^Leu mutant was shown to be highly resistant. Therefore, we concluded that the alkylation of the Met residues, introducing a permanent positive charge, was a major reason for the loss of CK activity.

## Conclusion

Our study confirms that Cys^283^ of rmCK as well as diverse Met residues were alkylated by SM. The use of IM^70^(-HETE)TV and KSM^179^(-HETE)TE as biomarkers for the verification of SM exposure, in addition to the already reported use of TC^283^(-HETE)PS (Steinritz et al. 2021), will be elaborated in future studies.

Alkylation of Cys^283^ was not suggested as primarily responsible for decreased rmCK enzyme activity, but, in contrast, the alkylation of Met^70^ and Met^179^ seemed to play a critical role in that context. Met residues were reported to be important for the conformational stabilization, high affinity ligand binding and function of proteins (Valley et al. 2012). Thus, it can be assumed that alkylation of Met-motifs in rmCK might cause a conformational change of rmCK. Initial experiments using the switchSENSE technique which allows determination of the hydrodynamic diameter (*D*_H_) of proteins (Blocquel et al. 2017; Cléry et al. 2017) were conducted to support our hypothesis (data not shown). The *D*_H_ of untreated wt-rmCK was determined to be 5.5 ± 0.16 nm while that of the alkylated wt-rmCK had increased to 7.67 ± 0.51 nm. The rmCK mutants exhibited a less prominent increase of *D*_H_ (6.5 ± 0.25 nm for Met^70^Ala, 5.65 ± 0.14 nm for Met^179^Leu, and 6.1 ± 0.23 nm for Met^70^Ala/ Met^179^Leu) suggesting smaller structural changes and thereby underlining the important role of Met^70^ and especially Met^179^ for stabilizing the rmCK protein structure. Additional crystallographic experiments were conducted (data not shown) to further prove the hypothesis of SM-induced conformational changes of the rmCK structure. The alkylated forms (wt and all mutants) exhibited a slower crystal growth than the non-alkylated forms, hinting towards a lower degree of conformational stability. Unfortunately, no adequate crystals of alkylated mutants suitable for synchrotron measurements were obtained, although various optimizations were applied. Future studies should thus investigate the protein structure of alkylated rmCK in more detail.

This is the first study showing that alkylation of Met residues by SM significantly impacts enzyme activity. It seems plausible that the activity of other enzymes might also be affected after alkylation of Met residues. This may help to understand the molecular toxicology of alkylating agents, especially SM, in more detail.

## Supplementary Information

Below is the link to the electronic supplementary material.

Supplementary file1 (PDF 211 KB)

## Abbreviations

a.u.: Arbitrary units
d3-atr: Three-fold deuterated atropine
ACN: Acetonitrile
ADP: Adenosine diphosphate
AGC: Automatic gain control
ATP: Adenosine triphosphate
CE: Collision energy
CK: Creatine kinase
Cr: Creatine
d8-SM: Eight-fold deuterated sulfur mustard
ddMS^2^: Data-dependent tandem-mass spectrometry
D_H_: Hydrodynamic diameter
DTT: Dithiothreitol
EDTA: Ethylenediaminetetraacetic acid
EGTA: Ethyleneglycoltetraacetic acid
ESI: Electrospray ionization
EtOH: Ethanol
FA: Formic acid
FWHM: Full width at half maximum
HEPES: 4-(2-Hydroxyethyl)-piperazine-1-ethanesulfonic acid
IAA: Iodoacetamide
IEX: Ion-exchange chromatography
IT: Injection time
K: Kelvin
µLC: Micro liquid chromatography
MS^2^: See MS/MS
MS/HR MS: High-resolution tandem-mass spectrometry
MS/MS: Tandem-mass spectrometry
NCE: Normalized collision energy
Ni-NTA: Nickel-nitrilotriacetic acid
NMR: Nuclear magnetic resonance
nt: Nucleotide
OD: Optical density
PBS: Phosphate-buffered saline
PCr: Phosphocreatine
PRM: Parallel reaction monitoring
ProtK: Proteinase K
RCF: Relative centrifugal force
rmCK: Rabbit muscle CK
RT: Room temperature
SM: Sulfur mustard
TB: Terrific broth
TEV: Tobacco etch virus
*t*_R_: Retention time
UF: Ultrafiltration
wt: Wildtype
