# Supplementary material for: Alkylation of rabbit muscle creatine kinase surface methionine residues inhibits enzyme activity in vitro
Source: Arch Toxicol. 2021 Aug 16;95(10):3253–61. doi: 10.1007/s00204-021-03137-6 (PMC8448711; doi:10.1007/s00204-021-03137-6)
Supplement: Supplementary file 1 — Supplementary file1 (PDF 211 KB) [file 204_2021_3137_MOESM1_ESM.pdf]

Supplement Material

Alkylation of rabbit muscle creatine kinase surface methionine residues inhibits enzyme activity *in vitro*

Dirk Steinritz, Robin Lüling, Markus Siegert, Harald Mückter, Tanja Popp, Peter Reinemer, Thomas Gudermann, Horst Thiermann, Harald John

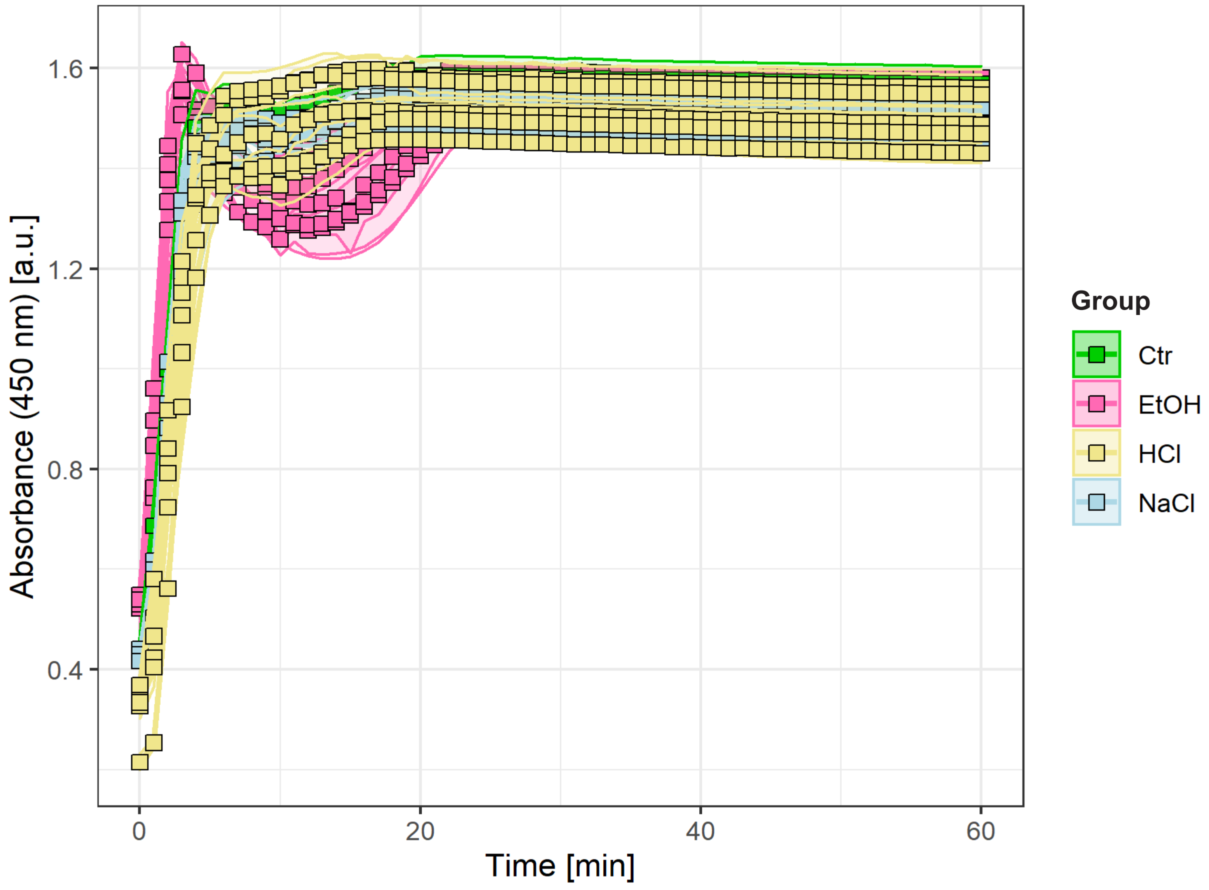

Supplement Figure S1: CK actitivity measurements in the presence of SM hydrolysis products or solvent. The solvent EtOH (2% (v/v), 0.2 mM H^+ or 8 mM Cl^-, the latter being hydrolysis products of SM, had no effect on CK activity at any pre-incubation time (0 - 30 min). Data are derived from 3 individual experiments per group and time (n = 3). Ribbons indicate the standard deviations.

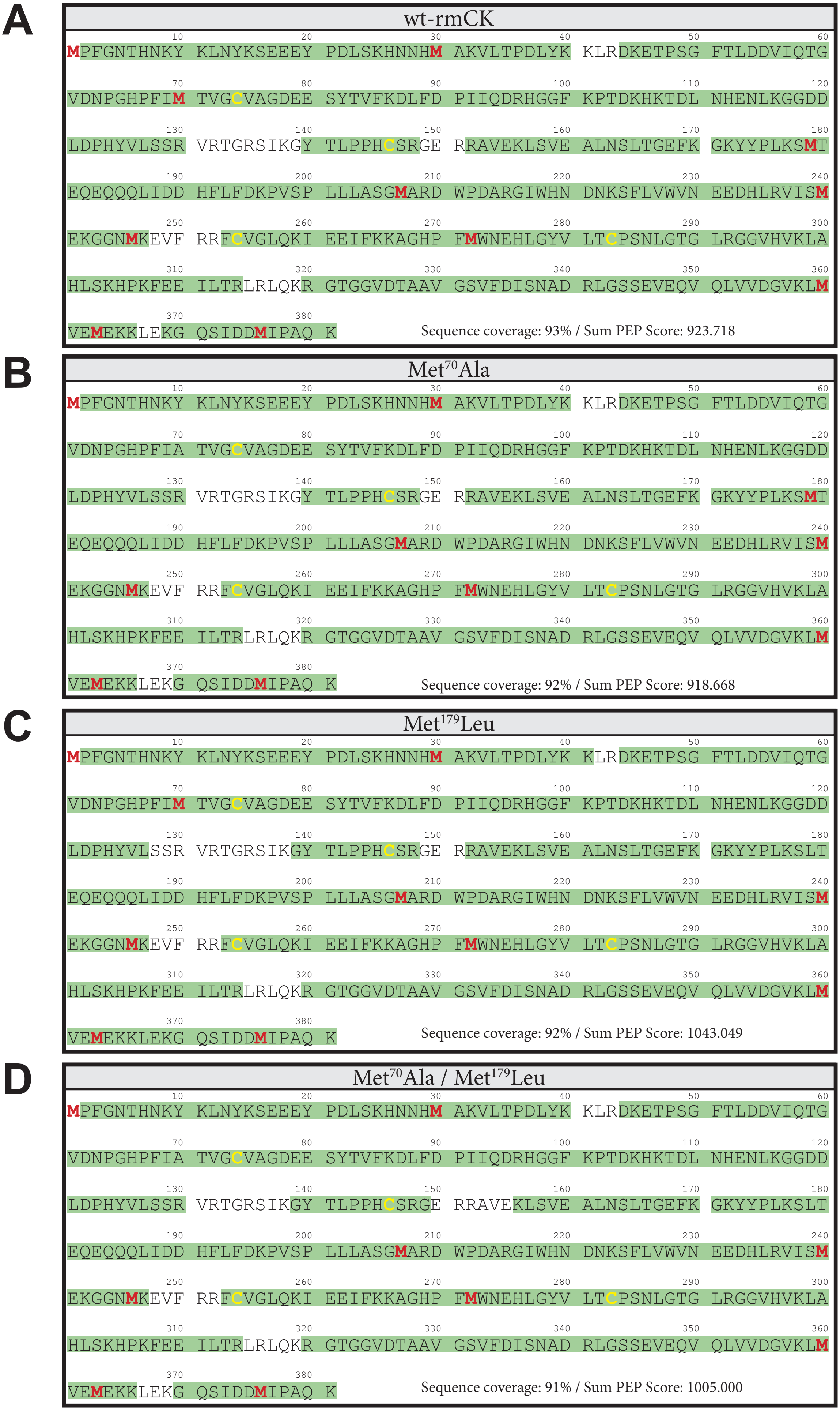

Supplement Figure S2: Schematic representation of peptide sequences matched in MSMS analysis after trypsin-mediated proteolysis for rmCK wildtype (A) and mutants (B-D). Methionines are highlighted in red, cysteines in yellow and green boxes represent identified peptides.

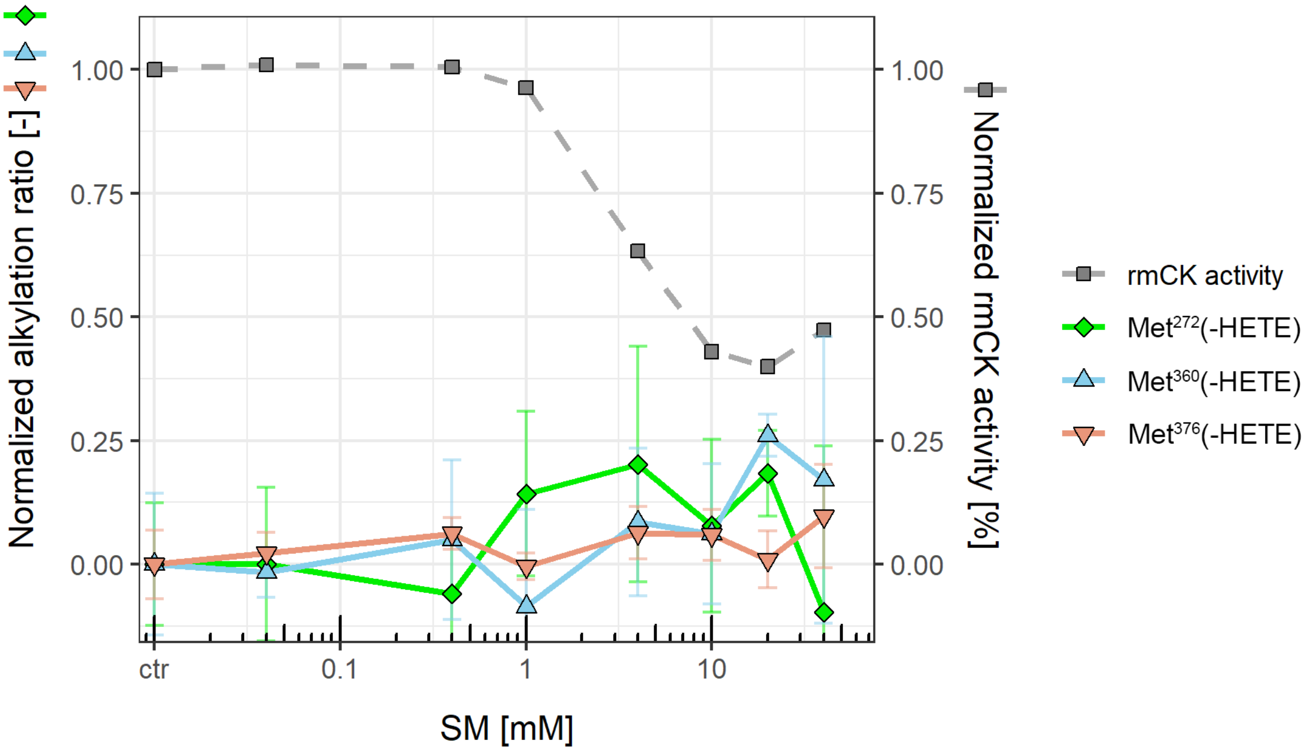

Supplement Figure S3: Correlation between alkylation ratio of Met^{272}, Met^{380}, Met^{376} and CK activity. rmCK was pre-incubated with SM for 15 minutes. The alkylation ratio was calculated by normalzing the peak area of the alkylated precursor ions to the peak area of the corresponding non-alkylated control precursor ions. Normalized CK activity was calculated by normalizing the AUC of the respective activity curves to control AUCs. A correlation between alkylation of Met^{272}, Met^{380}, Met^{376} and CK activity was not evident. Data are derived from 3 individual experiments (n = 3). Error bars represent standard deviation.
